# Dental Nomenclature

**Published:** 1899-04

**Authors:** S. D. Ruggles

**Affiliations:** Portsmouth, Ohio


### THE DENTAL REGISTER.

VOL. LIII.] APRIL, 1899. [No. 4.

### COMMUNICATIONS.

### Dental Nomenclature.

BY S. D. RUGGLES, D.D.S., PORTSMOUTH, OHIO.

Read at Ohio State Dental Society, December, 1898.

The substance of this article is taken from reports on nomenclature from several sources, and is not the result of original work. The subject was suggested by a leading member of our society as one of great importance and one sadly neglected by even the most active workers in the dental profession.

While examining reports a very interesting pamphlet, containing exactly the points wanted, was discovered, and through the kindness of Dr. Black permission was given to have it reprinted and distributed among the members of this society.

The history of dental nomenclature is much the same as that of other sciences, simple in the beginning, it gradually became more complex as advancement was made. In the early growth of any science the nomenclature is the product of a few individuals working along the lines in which they are most interested, and as new writers enter the field new words are coined. This makes it practically impossible to adopt a definite and unchangeable system so long as progress is being made.

The original nomenclature of botany and zoology was derived entirely from the Latin language or Latinized vernacular words. This is also the case with medicine, which at present has no fixed form.

The vernacular forms of speech are ever on the increase and are to a great degree the cause of much repetition of technical terms. It is impossible to adopt any one language to this work. For instance, if the Latin language were used, we have words without Latin equivalents, such as jaw, root, enamel, etc., and others that have been so thoroughly established by usage by our best authors that it would be very unwise to attempt a change.

What we need most is not a vast number of new terms, but to make ourselves familiar with the best ones already in use—systematize them, if you please. After this has been done, a combined effort should be made by all editors of dental journals to conform to the nomenclature adopted, and not use so many terms supposed to have the same meaning. This is the source of a great deal of our trouble.

It is a very common occurrence in dental societies for a member to resort to a black-board in order to make clear some minor point, such as the position or location on a given surface. We should be able to express our ideas accurately and in a concise manner, in a way in which our hearers will understand as well as ourselves.

So much attention is given operative dentistry that a definite plan for naming the surfaces of teeth and the different parts of cavities is of the utmost importance. This system should be one that could become international if possible, and if not possible, let it be national, so that a person reading a paper before a society other than the one of which he is a member will have some assurance that his efforts and ideas are not misinterpreted.

The object of this paper is not to suggest new terms but to urge the adoption of that system reported at the World's Columbian Dental Congress, which met in Chicago in 1893. From this a partial system, composed of thirteen nouns and ten adjectives, was selected by Dr. G. V. Black as being the most suitable names for the surfaces of teeth, cavities and parts of cavities. This group of twenty-three words constitutes by far the greatest number of technical expressions used by dentists, and are practically the same in English, French and German. This gives in reality an international nomenclature. The nouns are names with which we are all familiar, though perhaps not from a technical standpoint. This is also true of the adjectives.

In order that all may see I have written the entire list upon the board and will try to demonstrate as far as possible the use of those with which you are not so apt to be familiar.

As the black-board space was limited I have taken the ten adjectives and only three of the nouns to demonstrate the system.

There are five surfaces to all teeth, whether posterior or anterior, and are named according to their relative position to the other parts of the mouth. Take as an example the lower left first molar.(referring to figure 1). Consider the crown as a cube with the angles more or less rounded; the surface next to the tongue is called the lingual surface, that next to the cheek the buccal surface, that surface away from the median line following the curvature of the arch the distal surface, that toward the median line the mesial surface, while the surface in contact with the opposing teeth is the occlusal surface. This is true with the posterior teeth, while with the anterior teeth incisal edge takes the place of occlusal, and labial the place of buccal, as we have here the lips instead of the cheek.

The axial surface of a tooth is that surface parallel with the long axis of the tooth, and each tooth has four axial surfaces—mesial, distal, buccal and lingual.

The proximate surface is that surface that lies next to an adjoining tooth, but otherwise designates no particular one of such surfaces. It is very important that the V-shaped space formed by these surfaces should be maintained. This is the inter-proximate space.

This same nomenclature applies to the naming of cavities and parts of cavities. For instance, a cavity on the occlusal surface would be termed an occlusal cavity, while if it extended on to the mesial surface also it would be a mesio-occlusal cavity.

The walls of cavities are named according to the surfaces upon which they open or are situated, regardless of the outline of the cavity. In a mesio-occlusal cavity of a molar, we have the lingual wall next to the tongue, the buccal wall next to the cheek, the axial wall parallel with the axial surface upon which the cavity is located, while the wall toward the gum is called the gingival wall

That wall of occlusal cavities that is next the pulp is called the pulpal wall.

When speaking of the walls of cavities you include both dentin and enamel, while in the term margin, enamel only is implied.

A few words on angles will be sufficient. We have two kinds of angles—line and point angles. Two surfaces are required to form a line angle, for instance, the mesial and buccal surfaces join and form the mesio-buccal line angle, while by uniting with a third, the occlusal surface, we have formed a point angle—the mesio-bucco occlusal point angle.

I think this will demonstrate sufficiently the advantages of the system, and the great aid it will be in making ourselves better understood. Of course, all the terms and more are found in the little pamphlet, which I hope you will all read.

DISCUSSION.

Dr. H. T. Smith: A paper of this kind may appear at first to the dentist as more or less elementary; to those men who are teaching, it is of course the A. B. C.'s of filling teeth. The students are given these terms and taught these in the naming of cavities. It is their operating nursing-bottle, so to speak. I think these are mostly useful in the naming of cavities, in their keeping of records and the recording of operations in their ledgers and journals. I personally should be very glad to hear a discussion on that point, as it appears to me they are very useful to dentists in this respect. Of course, with the introduction of this system of nomenclature both in America and abroad, it would be necessary that the dentist should become familiar with this in order to properly read and write his papers and the reading of text-books. He cannot get along without it. The point brought up in reference to the "axial surface" is a very good one indeed, and the word pulpal being a new term might have a little further discussion or explanation. Dr. Ruggles referred the origination of the term to Dr. Black. I think that Dr. Clayton also put in a claim for the originality of the term. The point which Dr. Ruggles did not bring out was its use in proximal cavities, where the cavity is not compound and where a distinguishing term should be used between the pulpal wall and occlusal wall, the latter being in a proximate cavity; the wall at right angles to the long axis of the tooth and the pulpal wall being parallel with the long axis of the tooth. The term occlusal in that kind of cavity, that wall is usually cut down, as it should be, in making the cavity a compound one. Another very interesting point brought out in the little pamphlet which I have read is the fact that the French authority has found it necessary to come to America for the basis of his statements for nomenclature; so I think it is a very great credit to our own countrymen, and to our dentist in the West, Dr. Black. The pamphlet covers the ground so well that I have no nothing further to bring out. Mesial and distal, although somewhat new in dentistry, have been mentioned for some years in the works of Dunglison & Gould, medical dictionaries. So they have been used in medicine for some time, but are new in dentistry. This is so with many terms. That is about all I have to say. I think Dr. Ruggles is to be very much commended for his interest in getting out this little pamphlet for the use of the members of this society.

Dr. Butler: This matter of dental nomenclature is one which should interest us all if we wish to be estimated as professional men, not even mentioning scientific; so that if we go into any meeting and take part in discussion or express ourselves upon any subject we may be able to do it with intelligence, by the use of proper terms, so we need use no unnecessary phraseology in order to make ourselves understood, nor by taking a black-board and drawing a lot of diagrams and saying, I want you to understand I mean so and so, but by the use of terms understood by all should be sufficient. We had the credit, I think, of having done something in this direction in the old American Association, and I am very glad the matter has been brought up here in the Ohio State Society, and the preparation and putting into form what has been done up to the present time in this work, by Dr. Ruggles and distributed to us in these little pamphlets, ought to be of very great value to us and we should bear a very kindly feeling to Dr. Ruggles, and any one else who is studious enough to put his time into it to get it into shape, so we can get at it in as good or even better manner than we have here.

Dr. Barnes: I think Dr. Ruggles should be thanked for bringing this matter before the society, even though it be an elementary lesson. One feature of this subject which I think we have failed to grasp or realize is the value in our operative work. If we have a correct understanding of the names of the surfaces of teeth we are bound to produce a better form in our work—a better anatomical form. This develops as we become better acquainted with the terms. The newer men in the profession fully understand these terms because they have been taught in the colleges. Those of the older school are not so well acquainted with them because they were taught other terms, and so long as there appears in our books of reference and text-books such terms as fang, eye-tooth, wisdom tooth and terms of that character, so long will it be necessary for us to teach in the elementary way, and so I for one feel very grateful to Dr. Ruggles for instruction in this direction.

Dr. Harroun: This idea of introducing elementary work in our society I feel is the right thing. Then I think we should not get the idea we are getting to be old men, but are young and still growing—taking our A. B. C.'s as it were, and do not know it all. And at the same time I do believe we can all learn in the elementary departments just as much as in the advanced work. We are having experiences related from time to time in the advanced work in the way of filling, practical work, extracting teeth, etc., and are not so liable to become rusty. For that reason every gentleman should do some elementary work as a review, and I think we will be benefited. In this work under discussion, it has not been completed. Dr. Black has not done all; there is more yet to be classified, work for us all to do. There was one point which was not noticed in the paper, another term, and that is the proximal surfaces. We had our attention called to the inter-proximal spaces, but there is a point here which is in contact, which is not a space but is called "embrasure." This is the point beyond the space.

Dr. Ambler: We must have a standard in all things. We have standard dictionaries, and if we expect to have terms which will be adopted by all, we must have a standard to go by. We have a standing committee in the National Dental Association on Dental Nomenclature, and it would be my idea we should accept as a nomenclature their views and findings in the derivation, spelling and pronouncing of the terms. Now, if we are going to take their standard I do not think we can find any better time to commence than right now, and if we are going to take their word we must make one change in that list of adjectives given. That is, to have them end in "al" and the National Committee decided that their preference would be proximal instead of proximate, so we should have all corresponding. One other matter, and that is the pronouncing of "dentine" and "dentin." The committee has decided on plain "tin," the "e" has been dropped. This has been done by the foremost journals for some time.

Dr. Snyder: There is more dispute about the word proximal and proximate than any other term I can think of. Would it not be well to consider that one which is proper? Dr. Ruggles has said something about it, but would it not be well to consider that one point so we can talk with our patients correctly?

Dr. Molyneaux: The term as spoken of by Dr. Ambler was correct; "al" is considered the correct ending. I was on that committee and helped to make the report. That was the term ending as decided upon.

Dr. Stephan: I am very glad to hear so much of a discussion upon this paper. It is of great interest to all of us and more particularly to those connected with colleges and their work, because it enables us to teach exactly what should be taught and what we wish. It is a plain stand for better work. The time is coming when a system of nomenclature will be devised which will enable us to give exact instructions in operative work. There is no single system at present in the forming of cavities, and I think a uniformity in this line will be greatly assisted by this nomenclature; we will be better able to teach others how we form our cavities. After this we will be able to reduce these cavities to a graded system, and we will then really be scientific men and will have a scientific basis to work upon. I think this is a great step in advance, and while seemingly elementary, it is the very highest order of scientific work.

Dr. Cooper: I am only a young member in this society and have a little hesitancy in speaking, but there is one point which should be emphasized in the study—that the work is not to end here with us. We are to teach these terms to our patients, and we will find that very few of them will be familiar with the terms. There is where I find the greatest trouble. We are always obliged to explain the meaning of a term or to use a term which they understand. It seems to me it is better to use the correct term and explain it to them. This elementary work and system of education in that way reaches a great deal farther.

Dr. Taft: The points presented this morning are all very important and should be carefully studied and heeded by the profession, and especially should a correct method of naming prevail in our schools so that the younger members of the profession and those who are about to enter the same should have a correct nomenclature at least. They should also be impressed with the fact that much of the vague and defective namings which have been in common use should be avoided. And, perhaps, it is better to bring them at once upon a correct method, rather than try to compromise at all. There is a disposition with many to compromise in this matter; take the old and the new and mix them. I think the better method is to make a full and complete step by teachers, and writers as well. A point here that ought to be considered in taking up a subject of this kind is in introducing and impressing a new nomenclature on students; it has reference to their preliminary education. As has been stated in the paper this nomenclature is based largely upon the Latin language, so that one having a thorough knowledge of that language in the beginning of his student life and work will be far more likely to secure a grasp of this subject than one with no knowledge of the language at all. If he has no knowledge of Latin can he simply commit to memory the names without understanding the meaning of them and retain them? I regard this as a strong argument in favor of requiring all our student to study

Latin. This should be a requirement for entrance in every dental school. I know it is in very many of our schools a preliminary requirement, but in some it is not; it is absolutely overlooked. A student at first says: what is the use of a language which has been dead a hundred years, but yet he does not recognize the fact that our language is taken from it. After all, it does live in our own language. This is the point which should receive special attention. Now, in school and class-work you will find that very frequently the old and faulty names are used. That occurs, of course, with the older persons because of habit, having been trained that way in the beginning, and it is not an easy matter to overcome it. How very common it is to hear the teacher say, *dens sapientia*, canine tooth, etc. A great many editors and writers when they speak of cuspid say canine tooth, as you all know; and a great many other writers speak of a six-year molar. This reformation cannot be wrought in a day; I think everybody recognizes that fact, but let us not become impatient because a change is not made in a brief time. It grows, it is not created at once, but is a rather slow growth in our present condition. Let us remember that, and so far as we can, do everything possible to correct our nomenclature, and have it understood why we make the changes. If a name is advanced to-day over one used yesterday, why is the new better? Let every student understand this. A gentleman a few minutes ago referred to the education of the people on this subject, talking to them in our own nomenclature. While the dentist should do all in his power in this direction, yet he should not use a stilted language with which they know nothing. So let us be careful in that respect not to use a language they do not understand. I have occasionally heard dentists talking to their patients and using a language which the patient knew nothing about and it amounted as nothing to them. He might just as well have used Choctaw or Arabic. Always use the language which will be the medium of correct communication, and if the person addressed does not understand it, it amounts to nothing. The speaker might just as well have remained silent. Our language to our patients should be a very different one from that with which we address a class of students, especially so far as nomenclature is concerned. Let us use that with which we can convey the thoughts we have with the most force and clearness. Now, these faulty names which have been used so much ought to be corrected everywhere, especially to students. But let us remember in our writing and in our speaking, that the growth of a language is comparatively slow and not gather up a great array in this matter that will overwhelm the hearer, but give him just what he can digest and utilize in the future. Simply use it as rapidly, as judicially and as carefully as it can be taken and assimilated. The work that is being done by the National Association is a very good one, indeed. The profession owes Dr. Black a debt of gratitude, we can hardly appreciate how much he has done along this line, in the future we shall realize the importance of this subject more than we do to-day.

DR. W. D. SNYDER: In the discussion of this question I find that those having taken part were not all of one mind, but all agreed with the paper pretty well, with the exception of a little criticism on one point and that seems to be a difference in the pronouncing the terms of the subject in hand. Some pronounced it no′menclature and others pronounced it nomencla′ture, there is a difference in the accent. If the National Association has taken this matter up and appointed acommittee to revise this work, it strikes me we should all be posted on the proper pronunciation of this term, and I believe I have heard it more variously pronounced than any one word I know of, and I arise simply to a point of instruction, perhaps more than to discuss the question.

DR. TAFT: I think authorities differ in reference to its pronunciation. Both used, although I think the committee discussed that subject.

DR. MOLYNEAUX: The Century Dictionary was taken as reference, and the word was decided to be nomencla′ture. The other was used only by a very few people, and the work of the committee of the National Association suggested the Century Dictionary to be used as a standard.

DR. RUGGLES: I feel highly complimented, indeed, that I

have brought out so much discussion; I know it was elementary, but I am glad to see that so many are interested. In reference to the pronunciation of nomenclature I think the point well taken, and it should have been decided earlier in the discussion. Dr. Taft struck the key-note of this discussion when he said we should have few terms and be familiar with those used. That, I think, is the great advantage of this system. These ten adjectives can be applied in a hundred different ways. This system has been used in one of the largest schools in the country. Dr. Black's edition is used at present, and has seemed to be very successful indeed. It is also used in the East. The words proximal and proximate have been discussed time and time again, but the term proximal is incorrect, as used by dentists. I have the definition here, as I expected it to be brought up. I think Dr. Smith misunderstood me when I defined the term pulpal—as the pulpal wall is at a right angle to the long axis of the tooth, instead of being parallel with it, as he said. All adjectives should end in *al*. However, Dr. Black's latest work does not use it. The term used in my paper is the term used in his book. I would like to see some plan outlined or some standard given whereby we can be governed by this system, and make it a point to see that all papers submitted to the public use the correct nomenclature. We can correct more mistakes in that way than any other. I am very glad indeed you received the paper as you did, and thank you for your criticism.

Dr. Taft: I do not know whether it would be a feasible matter to make it compulsory on the editors to correct papers, etc. I have no doubt that editors, generally, will endeavor to conform to correct usages so far as they can. Of course, in the past they have been subject to infirmities as has every other person, but they are being rapidly improved.

Dr Molyneaux: The discussion is closed.

